# Diffuse Alveolar Hemorrhage Leading to Respiratory Failure in Mixed Phenotype Acute Leukemia: A Fatal Pulmonary Complication

**DOI:** 10.7759/cureus.89713

**Published:** 2025-08-10

**Authors:** Mariela Ginés-Rosario, Belissa Lopez-Pena, Zydnia Piñeiro, Maria C Perez-Mitchell, Ricardo Fernandez-Gonzalez

**Affiliations:** 1 Internal Medicine, San Juan City Hospital, San Juan, PRI; 2 Pulmonary and Critical Care Medicine, San Juan City Hospital, San Juan, PRI

**Keywords:** blood malignancy, diffusse alveolar hemorrhage, leukemia, mixed-phenotype acute leukemia, pulmonary complications

## Abstract

Mixed phenotype acute leukemia (MPAL) is a rare and diagnostically challenging hematologic malignancy characterized by the co-expression of myeloid and lymphoid lineage markers. We present the case of a 61-year-old woman with newly diagnosed MPAL who developed diffuse alveolar hemorrhage (DAH) shortly after induction chemotherapy. Despite aggressive supportive care, including bronchoscopy-guided diagnosis, high-dose corticosteroids, antifibrinolytics, and lung-protective ventilation with prone positioning, her condition rapidly deteriorated into multi-organ failure and death. This case highlights the need for early recognition of pulmonary complications in high-risk leukemias and underscores the limitations of current management strategies in the setting of DAH complicating MPAL.

## Introduction

Mixed phenotype acute leukemia (MPAL) is a rare and aggressive leukemia that accounts for only 2-5% of adult cases [1]. It is defined by the co-expression of myeloid and lymphoid markers on the same blast population, as outlined by the World Health Organization (WHO) [2]. MPAL can be further classified into B/myeloid or T/myeloid subtypes, with varying responses to treatment and prognostic implications [3,4]. Due to its rarity and diagnostic complexity, MPAL often lacks standardized treatment approaches and carries a poor prognosis. Pulmonary complications are well recognized in acute leukemias such as acute myeloid leukemia (AML) and acute lymphoblastic leukemia (ALL), where they may present as infections, leukemic infiltration, pulmonary edema, or diffuse alveolar hemorrhage (DAH) [5,6]. While DAH has been described in AML and ALL, its occurrence in MPAL is exceedingly rare, with only isolated reports in the literature [7,8]. This case is particularly notable for the rapid onset of DAH in a patient with newly diagnosed B/myeloid MPAL following induction chemotherapy. It illustrates the diagnostic and therapeutic challenges clinicians face when rare hematologic malignancies present with acute pulmonary deterioration. The absence of disease-specific guidelines for such complications highlights a critical need for awareness and multidisciplinary intervention.

## Case presentation

**Figure 1:**
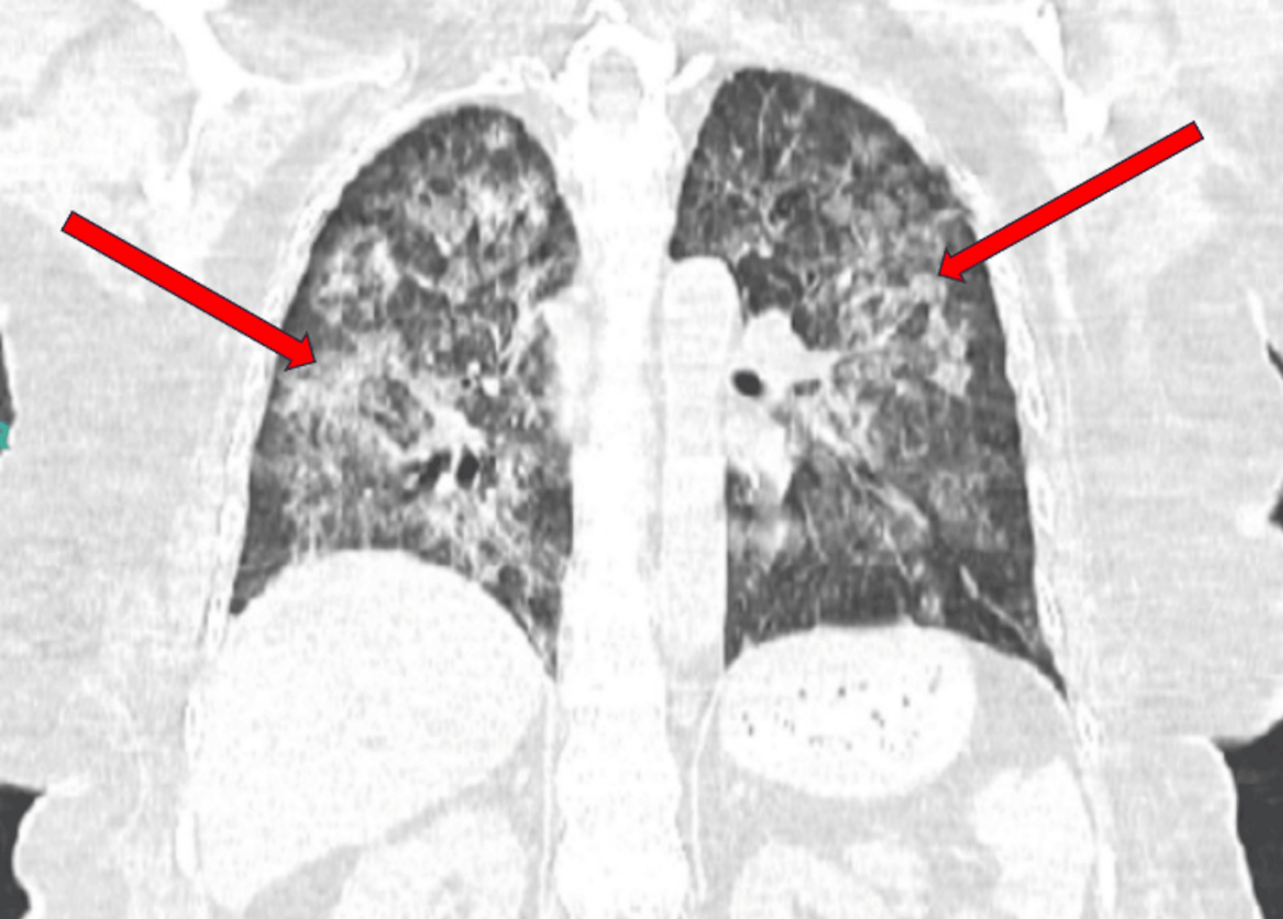
Coronal chest CT in lung window demonstrating diffuse, bilateral ground-glass opacities with patchy areas of consolidation throughout both lungs. Given the bronchoscopy findings of DAH, the CT findings represent bloody alveolar infiltrates. Red arrows indicating areas of ground-glass opacities. CT: Computed tomography, DAH: Diffuse alveolar hemorrhage

**Figure 2:**
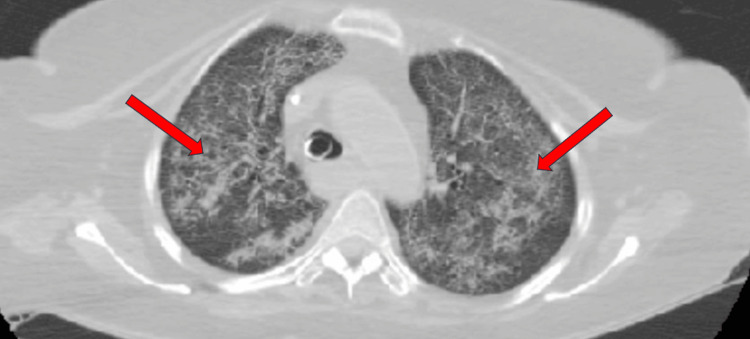
Axial chest CT in lung window demonstrating bilateral, diffuse ground-glass opacities with interspersed areas of consolidation, predominantly in the central and dependent lung regions. The pattern is symmetric and involves both upper and lower lobes. These findings are consistent with diffuse alveolar hemorrhage. Red arrows indicating widespread ground-glass opacities in both lungs, accompanied by scattered regions of consolidation.

**Figure 3:**
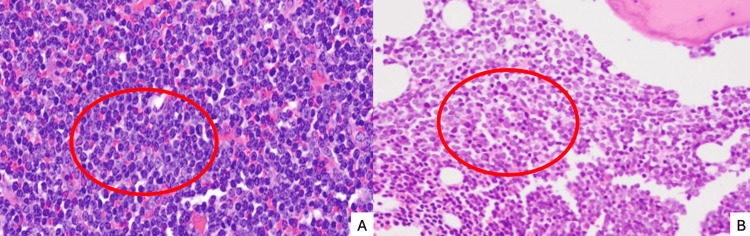
Hypercellular bone marrow histopathology. Red circles: areas showing dense infiltration of immature blasts and high mitotic/proliferative activity.

A 61-year-old woman with a past medical history of hypertension, hyperlipidemia, and hypothyroidism treated with levothyroxine 75 mcg daily presented to the emergency department with an acute onset of progressive dyspnea, fatigue, night sweats, and unintentional weight loss over the preceding month. She denied fever, cough, or hemoptysis. On physical examination, she was tachypneic (respiratory rate: 28 breaths/min), hypoxic (SpO₂ 85% on room air), and tachycardic (heart rate: 112 bpm). Cervical and supraclavicular lymphadenopathy was noted, and diffuse inspiratory crackles were heard bilaterally. Initial laboratory tests showed a white blood cell count of 98,000/µL with 77% blasts, hemoglobin of 8.4 g/dL, and a platelet count of 32,000/µL (Table 1). D-dimer was significantly elevated at 4,280 ng/mL (Table 1). Peripheral smear showed abundant smudge cells, occasional basophilic stippling, and fragile basket cells, suggestive of blast fragility and high tumor burden. Chest computed tomography (CT) in lung window (Figures 1, 2) demonstrated bilateral ground-glass opacities and patchy consolidations. Bone marrow biopsy confirmed a hypercellular marrow (Figure 3), with immature infiltrate and blasts expressing myeloperoxidase (MPO) and terminal deoxynucleotidyl transferase (TdT), consistent with B/myeloid MPAL. Induction chemotherapy was initiated using vincristine, prednisone, daunorubicin, and cyclophosphamide, along with hydroxyurea for cytoreduction and allopurinol for tumor lysis syndrome prophylaxis. Within 48 hours, the patient developed progressive hypoxemia and was intubated for acute respiratory distress syndrome (ARDS). Hemoptysis prompted bronchoscopy, which confirmed diffuse alveolar hemorrhage (DAH), with progressively bloody return on bronchoalveolar lavage (BAL). The BAL cytology report showed numerous red blood cells (RBCs) and abundant hemosiderin-laden macrophages (confirmed by Prussian blue stain), findings consistent with diffuse alveolar hemorrhage (DAH) in the context of MPAL. High-dose methylprednisolone, aminocaproic acid, and lung-protective ventilation with increased positive end-expiratory pressure (PEEP) and low tidal volumes were administered. Despite prone positioning, her respiratory status worsened. Lactic acid was elevated (6.2 mmol/L). Creatinine increased from 1.1 to 2.1 mg/dL within 48 hours, and urine output declined (0.3 cc/hr). Platelet count dropped to 12,000/µL, and INR increased to 2.3, consistent with disseminated intravascular coagulation. She developed shock requiring norepinephrine and vasopressin therapy. Despite best efforts, she continued to decline due to refractory hypoxemia and multiorgan failure. After discussion with the family, comfort measures were initiated as per their wishes, and the patient died shortly after. 

**Table 1:** Laboratory test results upon admission.

Laboratory Parameter	Initial Laboratory Tests Values	Laboratory Reference Range
White Blood Cells	98,000 uL	4,800 to 10,800 uL
Hemoglobin	8.4 g/dL	12 to 14 g/dL
Platelets	32,000 uL	150 to 450 uL
Uric Acid	7.1 mg/dL	4.4 to 7.6 mg/dL
Lactate Dehydrogenase	352 IU/L	114 to 192 IU/L
Blasts	77%	0%
D Dimer	4,280 ng/mL	<500 ng/mL

## Discussion

DAH is an uncommon but devastating pulmonary complication in acute leukemia, most frequently reported in AML, where leukostasis, thrombocytopenia, and coagulopathy are common [5]. Its occurrence in MPAL is rare, with only limited case reports describing such presentations [8]. The pathogenesis of DAH in leukemias is multifactorial and includes thrombocytopenia (typically <20,000/μL), direct leukemic infiltration disrupting alveolar-capillary membranes, chemotherapy-induced pulmonary endothelial damage, and cytokine release syndrome that increases vascular permeability [6,8]. In this case, the patient presented with profound thrombocytopenia, elevated blasts, and cytokine-driven inflammation following chemotherapy, all likely contributing to the development of DAH. Management of DAH in leukemic patients is largely supportive and involves reversing coagulopathy and anti-inflammatory strategies. Platelet transfusions to maintain platelet counts >50,000/μL, antifibrinolytics such as aminocaproic acid, and high-dose corticosteroids are commonly used, although evidence for their efficacy is largely observational [6,8]. Recombinant activated factor VIIa has been proposed as a salvage therapy for refractory cases, though data are limited. Ventilation strategies must focus on minimizing ventilator-induced lung injury. Lung-protective ventilation with low tidal volumes, high PEEP, and permissive hypercapnia is recommended. Prone positioning has been shown to improve oxygenation in ARDS, although its effectiveness in DAH is less clear given the alveolar flooding [7,8]. Advanced support such as extracorporeal membrane oxygenation (ECMO) may be considered in centers where available. In this case, ECMO was not offered due to institutional limitations. Although there is no definitive evidence for ECMO efficacy in DAH secondary to hematologic malignancy, case series suggest it may serve as a bridge to recovery in select patients without active bleeding [8]. This case demonstrates how DAH in MPAL can rapidly progress despite standard interventions. The rarity of MPAL and its associated complications underscores the importance of early recognition, rapid diagnostic evaluation, and coordinated multidisciplinary management involving hematology, pulmonology, critical care, and infectious disease specialists.

## Conclusions

Diffuse alveolar hemorrhage in the setting of mixed phenotype acute leukemia represents a rare but catastrophic complication with limited treatment options and high mortality. The rapid onset of respiratory failure following induction chemotherapy underscores the vulnerability of this patient population to life-threatening complications. Despite timely diagnosis and multidisciplinary supportive care, outcomes may remain poor, particularly in settings without access to advanced therapies such as ECMO. Diffuse alveolar hemorrhage (DAH) remains a devastating, life-threatening complication in patients with hematologic malignancies, particularly in the setting of hematopoietic stem cell transplantation (HSCT). Our report adds to the existing literature by highlighting the occurrence of DAH in the context of mixed-phenotype acute leukemia (MPAL), a rare and biologically complex subtype of acute leukemia. Given the paucity of data on pulmonary complications in MPAL, our findings underscore the need for heightened clinical vigilance and early multidisciplinary management in this population. By documenting this association, we expand the understanding of DAH as a critical complication in MPAL, reinforcing the importance of timely recognition and intervention to improve outcomes in these high-risk patients.
